# Optical Intensities of Different Compartments of Subretinal Fluid in Acute Vogt-Koyanagi-Harada Disease

**DOI:** 10.1371/journal.pone.0149376

**Published:** 2016-02-12

**Authors:** Dusheng Lin, Xiaohong Luo, Lingying Meng, Guihua Zhang, Weiqi Chen, Haoyu Chen

**Affiliations:** 1 Joint Shantou International Eye Center, Shantou University and the Chinese University of Hong Kong, Shantou, Guangdong, China; 2 Shantou Center Hospital, Shantou, Guangdong, China; Justus-Liebig-University Giessen, GERMANY

## Abstract

**Purpose:**

To investigate the optical intensity in different compartments of subretinal fluid in acute Vogt-Koyanagi-Harada (VKH) disease by using spectral domain optical coherence tomography (SD-OCT).

**Methods:**

Fifty acute VKH eyes and 25 cases with acute central serous chorioretinopathy (CSCR) were included in this retrospective comparative study. The optical intensities of subretinal fluid, vitreous humour and the entire scanned region displayed by SD-OCT were measured with Image J by three independent readers. In the VKH eyes with subretinal septa, the subretinal fluid was segmented into two types of compartments, supra-septa space and sub-septa space. Optical intensity ratios of different compartments of subretinal fluids divided by vitreous humour or the entire scanned region were compared.

**Results:**

The measurement of optical intensity was highly reproducible (intraclass correlation coefficient> 0.9). The optical intensity of the supra-septa space divided by the vitreous humour was significantly higher compared to that of sub-septa space in VKH (mean difference = 4.27 ± 5.15, p <0.001). The optical intensity ratio of the supra-septa space (1.14 ± 0.12), but not subsepta space (1.05 ± 0.05) in VKH, was significantly higher compared to that of the subretinal space in VKH without the subretinal septa (1.07 ± 0.08), and the subretinal fluid in CSCR (1.08 ± 0.09). Similar results were found for the optical intensity ratios divided by the entire scan region.

**Conclusion:**

The optical intensity in the supra-septa space of VKH is higher compared to the sub-septa space in VKH, subretinal space in VKH and CSCR, suggesting that the components in these spaces are different.

## Introduction

Vogt-Koyanagi-Harada (VKH) disease is a common cause of bilateral panuveitis in darkly pigmented races. There is often involvement of other organs, including the ears, skin, and meninges.[1] The acute phase of VKH disease is characterized by diffuse choroidal inflammation and serous retinal detachment at the posterior pole.[2] With the introduction of optical coherence tomography (OCT), several microstructural changes have been identified in VKH disease, such as subretinal fluid, choroidal and retinal pigment epithelial (RPE) fold, fluctuation of the internal limiting membrane, subretinal septa, bulge of RPE, increased retinal thickness and choroidal thickness.[3–9]

In some cases, the subretinal space is separated into several compartments by subretinal septa on the cross sectional images of OCT.[10] There are arguments concerning the nature of these septa and compartments. At first, it was assumed that the septa are part of the outer retina and the supra-septa space is intra-retinal fluid [3], intra-retinal edema [11], or cystoid space [10]. Later, by using spectral domain OCT (SD-OCT), the septa was found to be continuous with the line representing the junction of the photoreceptor inner and outer segments (IS/OS) in the attached areas of the retina, suggested a splitting off of outer segments from the inner segments of the photoreceptor layer, and formation of a membranous structure through binding of these outer segments to each other, forming cysts in the supra-septa space within photoreceptor layer.[8, 12, 13] However, this hypothesis was questioned that intraretinal cysts or photoreceptor cleavage and vision cannot recover so rapidly or completely.[14, 15] On FFA, the subretinal compartments correspond to multilobular dye pooling, and there is no petaloid pattern. Therefore, it is assumed that the septa are fibrinous or proteinaceous subretinal exudates, whereas the supra-septa fluid is subretinal fluid segmented by a fibrous membrane.[16]

The purpose of this study is to investigate the component of fluids in different compartments of subretinal spaces in VKH by quantitatively comparing the optical intensities in these compartments on OCT images.

## Patients and Methods

### Subjects

This is a retrospective comparative study. The Institutional Review Board of the Joint Shantou International Eye Center approved this study and waived the informed consent due to the retrospective nature of this study. Our study complies with the Declaration of Helsinki. The patient records/information was anonymized and de-identified prior to analysis.

The patient database of Joint Shantou International Eye Center, Shantou University and the Chinese University of Hong Kong, from January 2012 to July 2013, was searched. The medical charts of patients diagnosed as having VKH disease or central serous chorioretinopathy, which was included as control, received further review. The patients received comprehensive ophthalmic examinations including best-corrected visual acuity (BCVA), intraocular pressure, slit lamp biomicroscopy of the anterior segment and retina with mydriasis, fundus fluorescein angiography and SD-OCT.

Diagnosis of VKH was based on the criteria defined by the International Committee in 2001 [1]. Diagnosis of acute CSCR was based on typical angiographic findings of focal neurosensory retinal detachment or retinal pigment epithelium (RPE) detachment with leakage at the level of the RPE, as seen on fluorescein angiography [17] without any sign of other diseases, such as uveitis, polypoidal choroidal vasculopathy, choroidal neovascularization, optic disc pitting or retinal vasculopathy. Patients with symptoms lasting longer than 3 months were excluded in this study.

### Spectral domain optical coherence tomography

SD-OCT examinations were performed with a Cirrus HD-OCT (Carl Zeiss, Germany). The macula was scanned with the standard fovea-centered five line raster scan protocol. The single B-scan that contained the largest volume of subretinal fluid was chosen. The grayscale images were exported as bitmap files, which is an uncompressed format. No further image manipulation was performed on the OCT data. Only the OCT image with an signal strength ≥6 were included according to the manual of Cirrus OCT.

### Optical intensity measurement

**Fig 1:**
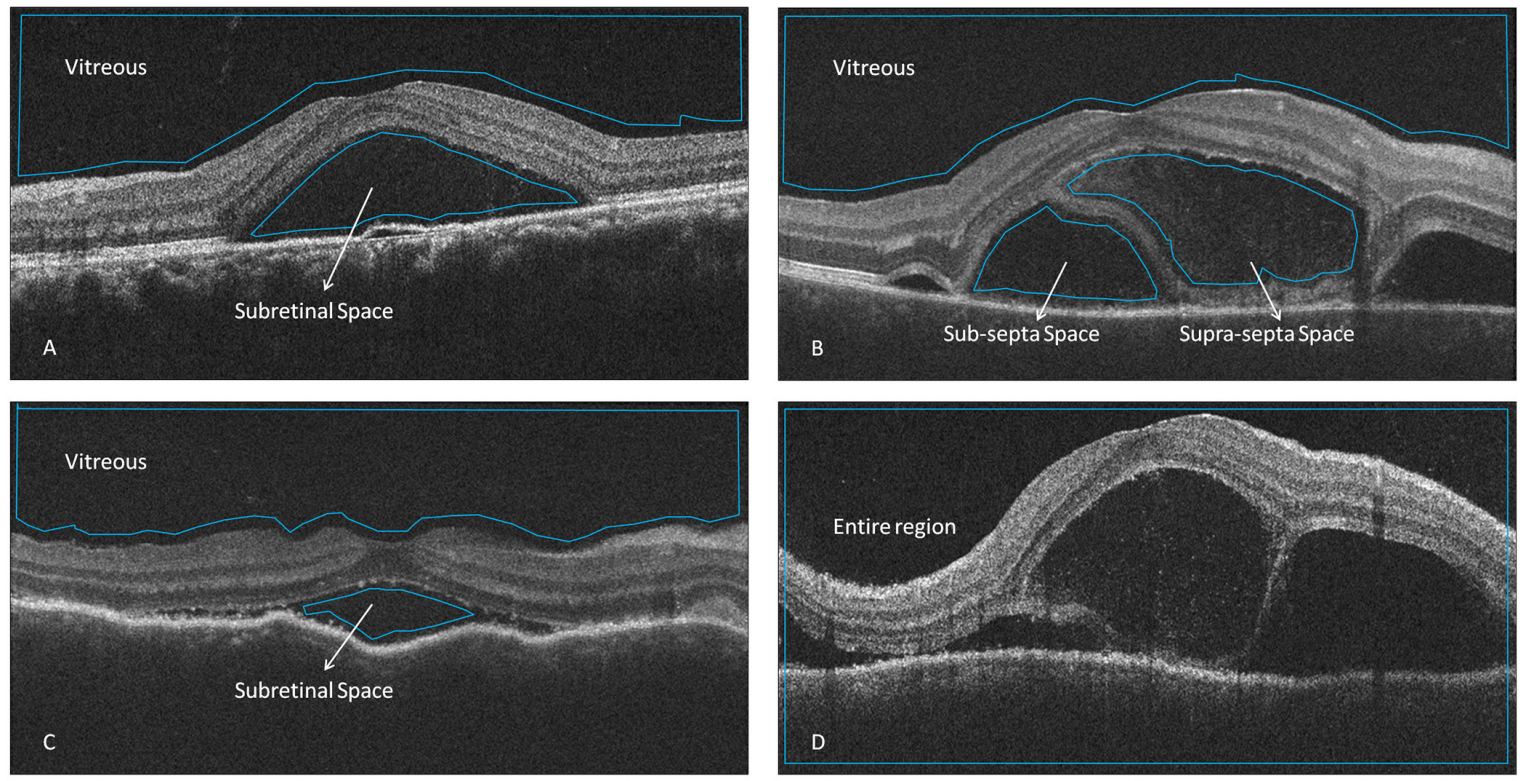
Selection of regions of interest on the optical coherence tomography images of Vogt-Koyanagi-Harada (VKH) disease and central serous chorioretinopathy (CSCR). A. selection of vitreous and subretinal space in CSCR; B. selection of vitreous, supra-septa space and sub-septa space in VKH with subretinal septa; C. selection of vitreous and subretinal space in VKH without subretinal septa; D. selection of entire region.

Optical intensity measurements were carried out with Image J software (National Institutes of Health, Bethesda, MD; available at http://rsb.info.nih.gov/ij/index.html) with a method modified from the literature.[18, 19] Four different types of regions of interest were defined with the largest possible area, including the supra-septa space (Fig 1A and 1B.) and sub-septa space (Fig 1B.) in VKH eyes with subretinal septa, subretinal space in CSCR and VKH eyes without subretinal septa, vitreous humour, and the entire B scan region (Fig 1C). The mean pixel intensities were calculated by the Image J software. Interpreting the exported B scan picture as 8-bit grayscale images resulted in 256 levels of gray, ranging from 0 to 255.To test the intra-observer reproducibility, the measurements were performed three separate times by Reader 1 (D.L.). To test the inter-observer reproducibility, the measurements were performed by three independent masked readers (D.L., M.L. and X.L.).

### Statistical analysis

The software SPSS (version 17.0; SPSS, Inc., Chicago, IL) was used to conduct statistical analysis. The intraclass correlation coefficients (ICC) were determined by a two-way mixed effects model for measurements of absolute agreement. The optical intensity is affected by many factors, such as OCT signal strength and media opacity. We calculated the optical intensity ratio of the subretinal space divided by the vitreous humour [18, 19]or the entire scanned region [20] for normalization. The optical intensity ratio of the supra-septa or sub-septa space in VKH eyes with subretinal septa was compared to that of the subretinal space in VKH eyes without subretinal septa and CSCR eyes by one-way analysis of variance and the Student-Newman-Keuls test. The optical intensities of sub-septa and supra-septa space in the same eyes were compared by a paired t-test because they were affected by the same level of signal strength or media opacity. Pearson correlation between optical intensity ratios and the best-corrected visual acuity at presentation or at the last follow-up was assessed. P values of less than 0.05 were considered statistically significant. SPSS (version 22.0, IBM Inc. Chicago, IL) software was used to perform statistical analysis and Sigmaplot (version 12.0, Systat Inc. Chicago, IL) software was used to draw statistical plots.

## Results

In total, 50 eyes of 30 patients with acute VKH and 25 eyes of 25 patients with acute CSCR were included in this study. The demographic information and optical intensity of each included subjects were listed in S1 Table. Ten of 85 eyes were excluded because image quality was unacceptable (signal strength < 6). Table 1 shows the demographic information, including age, gender and BCVA of the included subjects. There was a predominance of men in the CSCR group, but not in the VKH group (82.1% in CSCR vs. 46.7% in VKH, p = 0.004, chi square test). There was no significant difference of age of patients between the two diseases (p = 0.847, Student’s t test). BCVA was significantly worse in VKH than in CSCR group (0.77± 0.42 vs. 0.32 ± 0.23 LogMAR, p < 0.001, Student’s t test). Serous retinal detachment presented in all cases with VKH and all cases with CSCR, whereas subretinal septa only presented in 70% (35/50) cases with VKH, but not in CSCR. The measurement of optical intensities had high intra-observer repeatability and inter-observer reproducibility in all regions (all ICC > 0.927, Table 2).

**Table 1:** Demographic and clinical data of included subjects.

Variable	VKH	CSCR	P value	Statistics
N	30	25		
Age, year (range)	43.5±11.9 (21–76)	43.1±6.1 (25–62)	0.847	Student’s t test
Gender (male: female)	14:16	21: 4	0.004	Chi-square test
BCVA (LogMAR)	0.77±0.42	0.32±0.23	<0.001	Student’s t test

BCVA and age is presented as mean ± standard deviation. VKH: Vogt-Koyanagi-Harada disease; CSCR: central serous chorioretinopathy; BCVA: best corrected visual acuity; LogMAR: logarithm of the minimum angle of resolution

**Table 2:** Repeatability and reproducibility of optical intensity measurements.

	Intra-observer ICC (95%CI)	Inter-observer ICC (95%CI)
Vitreous	0.999 (0.998–1.000)	0.993 (0.986–0.997)
Sub-septa fluid in VKH	0.997 (0.995–0.998)	0.992 (0.984–0.996)
Supra-septa fluid in VKH	0.994 (0.986–0.996)	0.927 (0.836–0.974)
Subretinal fluid in VKH	0.999 (0.998–1.000)	0.907 (0.797–0.965)
Subretinal fluid in CSCR	1.000 (1.000–1.000)	0.945 (0.896–0.973)
entire region	1.000 (1.000–1.000)	0.981 (0.964–0.991)

ICC, intraclass correlation coefficient; CI, confidence interval; VKH: Vogt-Koyanagi-Harada disease; CSCR: central serous chorioretinopathy.

**Fig 2:**
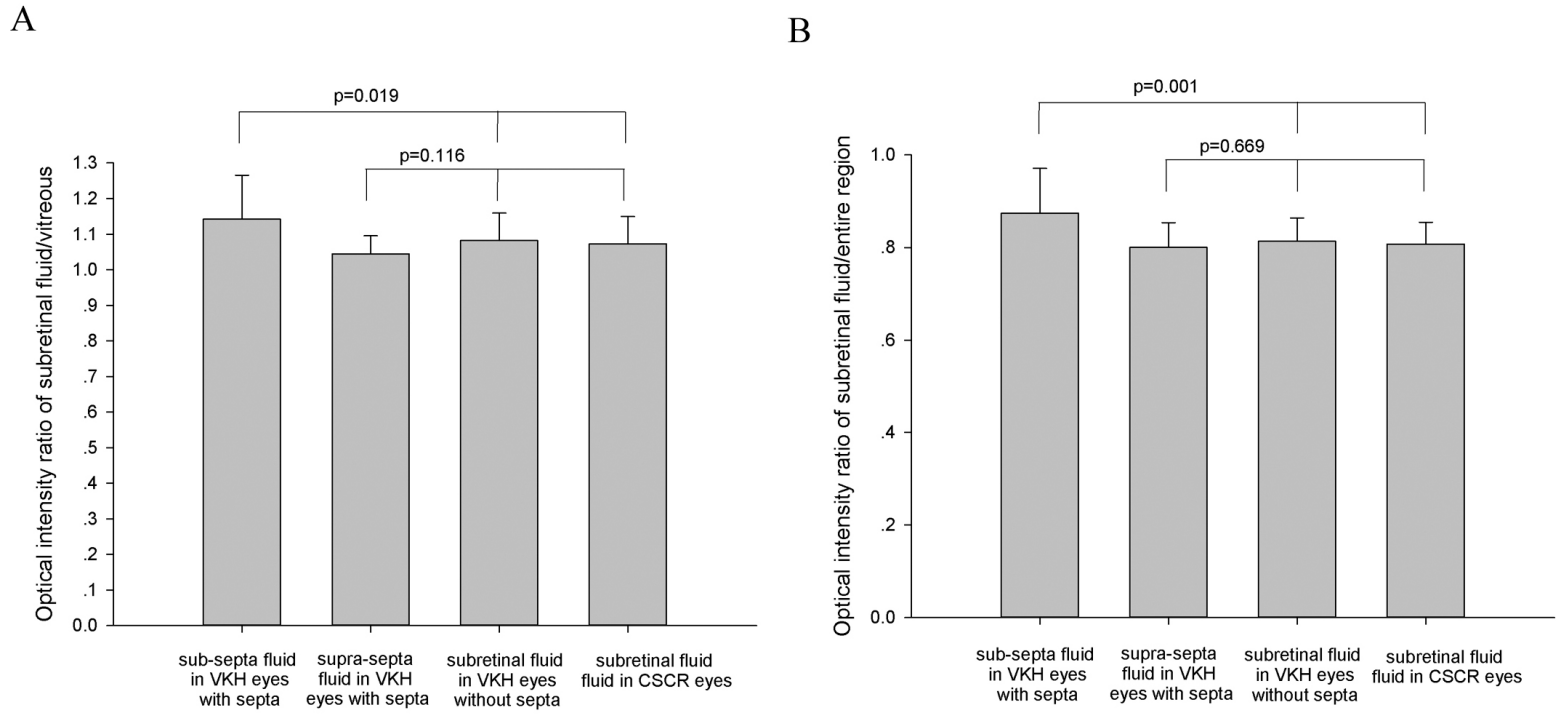
Comparison of optical intensity ratio in different compartments of subretinal fluids. A. optical intensity ratio of subretinal fluid divided by vitreous humour; B. optical intensity of subretinal fluid divided by the entire scanned region. The *p* value was calculated using one-way analysis of variance. VKH: Vogt-Koyanagi-Harada disease. CSCR: central serous chorioretinopathy.

**Fig 3:**
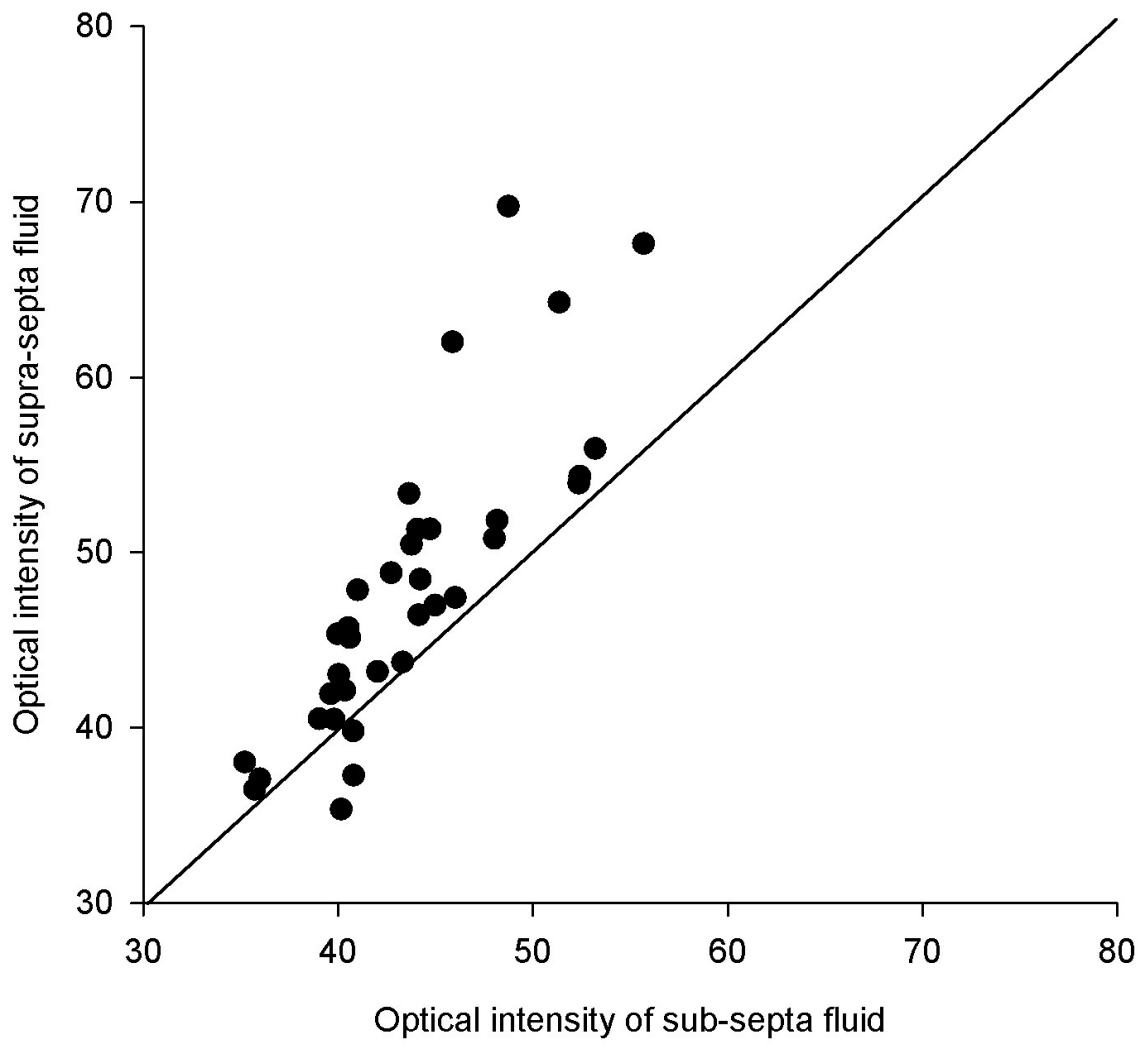
Scatterplot of the optical intensities of the supra-septa space and sub-septa in the same eyes with acute Vogt-Koyanagi-Harada disease. Most of the spots were above the 45-degree lines, indicating that the optical intensity of the supra-septa space was higher than that of the sub-septa space.

The optical intensity of supra-septa space was significantly higher compared to that of sub-septa space in VKH (mean difference = 4.27 ± 5.15, p <0.001, paired t test, Fig 2). The optical intensity ratio of supra-septa space divided by vitreous humour in VKH eyes (1.14 ± 0.12) was significantly higher compared to that of the subretinal space in VKH without subretinal septa (1.07 ± 0.08), and the subretinal fluid in CSCR (1.08 ± 0.09), with p = 0.019 (Fig 3A). There was no statistically significant difference between the sub-septa space in VKH with subretinal septa (1.05 ± 0.05) and subretinal space in VKH without subretinal septa or CSCR (p = 0.116, Fig 3A). Similarly, the optical intensity ratio of supra-septa space divided by the entire scanned region in VKH (0.87 ± 0.97) was significantly higher compared to that of the subretinal space in VKH without subretinal septa (0.81 ± 0.05), and the subretinal fluid in CSCR (0.81 ± 0.05) (p = 0.001, Fig 3B). There was no statistically significant difference between the sub-septa space in VKH with subretinal septa (0.80 ± 0.05) and subretinal space in VKH without subretinal septa or CSCR, with p = 0.669 (Fig 3B). There is no statistical significant correlation between the optical intensity ratios and the best-corrected visual acuity at presentation or at the last follow-up (all p > 0.05).

## Discussion

In this study we quantitatively investigate the optical intensity of different compartments of the subretinal space in VKH, using a measurement with high repeatability and reproducibility. The results show that the optical intensity of supra-septa space in VKH is higher than that of the sub-septa space in VKH with subretinal septa, subretinal space in VKH without septa or CSCR. There is no statistical difference in the optical intensity of sub-septa space in VKH with septa, sub-retinal space in VKH without septa and CSCR.

OCT is not only valuable in providing morphological images, but also quantitative measurement of the optical intensity (also referred to as optical density, reflectivity) of normal and pathological tissue. It has been reported that the optical intensity of the intraretinal or subretinal spaces with exudation, such as diabetic macular edema, retinitis pigmentosa, and CSCR, are higher than that of the vitreous humour. While the optical intensity of the intraretinal or subretinal spaces without exudation, including cone dystrophy and idiopathic perifoveal telangiectasia are lower compared to that of vitreous humour,[21] it has been shown that the optical intensity ratio of the subretinal space divided by the vitreous humour is significantly higher in patients with age-related macular degeneration, diabetic macular edema, CSCR, and postoperative macular edema, than in those with retinal detachment and retinoschisis.[19] In another study, it was reported the optical intensity ratio is not only increased in age-related macular degeneration, but also correlates with BCVA after anti-angiogenic therapy.[18] Furthermore, the optical intensity of intraretinal or subretinal space in diabetic macular edema is found to be correlated with visual acuity [22], fluorescein pooling,[23] and intravitreal concentration of vascular endothelial growth factor[24].These results suggest that the optical intensity of the intraretinal or subretinal spaces can be used as a biomarker and provide insight to the pathogenesis of retinal diseases.

Although the composition of the intra/sub-retinal spaces is still not well understood, a higher optical intensity signal may be an indication of increased component of the fluids being scanned, whereas a low optical intensity signal reflects a relatively clear fluid composition with fewer particles.[21] In current study, no significant difference in optical intensity is found between the sub-septa space or subretinal space between VKH and CSCR. The subretinal fluid of both VKH and CSCR are exudates from the choroidal vasculature through the breakdown of retinal pigment epithelium. Therefore, the composition of subretinal fluid coming from the serous retinal detachment space in these two diseases might be similar. So it is not surprising to find similar optical intensity of serous retinal detachment space in the two diseases.

The prevalence of subretinal septa in acute VKH has been reported to be 40–43% by time domain OCT [3, 10], and 85% by spectral domain OCT.[12]In our study, subretinal septa was found in 70% VKH eyes with spectral domain OCT, which is consistent with the literature. On the OCT image of VKH, the supra-septa fluid seems to be mildly reflective, whereas areas of sub-septa fluid are not reflective.[3] However, this result is based on qualitative observation. Our study quantitatively measures the optical intensity for VKH and finds that the optical intensity of the supra-septa space is higher compared to that of the sub-septa space.

The nature of the subretinal septa and the components of the supra-septa space fluid in acute VKH remains unknown and controversial. There are two hypotheses. In the first, the septa result from a splitting off of outer segments, from the inner segments of the photoreceptor layer, and formation of a membranous structure through the binding of these outer segments to each other, while the supra-septa space forms cysts within the photoreceptor layer.[8, 12, 13] The other hypothesis states that the septa are fibrinous or proteinaceous subretinal exudates that form on the RPE. Additional serous exudation through the RPE detaches the fibrin membrane from the RPE, forming septa that divide the subretinal space into multiple compartments.[16] Our study finds that the optical intensity in the supra-septa space is significantly higher compared to that of sub-septa space, suggesting that components of the supra-septa fluid are different from sub-septa fluid, and there are more particles in supra-septa fluid. This finding supports the hypothesis that subretinal septa is splitting off of outer segments from the inner segments, possibly due to the death of photoreceptors results in cell debris increased in the supra-septa space. Our study does not support the hypothesis that fibrinous or proteinaceous subretinal exudates dividing the subretinal space into multiple compartments, which should have similar optical intensity in this case.

Our study is a retrospective study and there may be sampling bias. A prospective study including more subjects is needed to confirm our results. In addition, there is only one time point in our study. Further study with longitudinal follow-up is needed to investigate the change in optical intensity of subretinal fluid in response to treatment. In summary, the present study finds significantly increased optical intensity in the supra-septa space compared to sub-septa space in VKH, subretinal space in VKH and CSCR, suggesting that the components in these spaces are different.

## Supporting Information

S1 Table Demographic and OCT characters of included subjects. (XLS)

## References

[1] Read RW, Holland GN, Rao NA, Tabbara KF, Ohno S, Arellanes-Garcia L, et al Revised diagnostic criteria for Vogt-Koyanagi-Harada disease: report of an international committee on nomenclature. Am J Ophthalmol. 2001;131(5):647–52. . 11336942 10.1016/s0002-9394(01)00925-4

[2] Rubsamen PE, Gass JD. Vogt-Koyanagi-Harada syndrome. Clinical course, therapy, and long-term visual outcome. Arch Ophthalmol. 1991;109(5):682–7. Epub 1991/05/01. . 2025171 10.1001/archopht.1991.01080050096037

[3] Maruyama Y, Kishi S. Tomographic features of serous retinal detachment in Vogt-Koyanagi-Harada syndrome. Ophthalmic surgery, lasers & imaging: the official journal of the International Society for Imaging in the Eye. 2004;35(3):239–42. . 15185793

[4] Gupta V, Gupta A, Gupta P, Sharma A. Spectral-domain cirrus optical coherence tomography of choroidal striations seen in the acute stage of Vogt-Koyanagi-Harada disease. American journal of ophthalmology. 2009;147(1):148–53 e2. Epub 2008/10/07. 10.1016/j.ajo.2008.07.028 . 18834577

[5] Kato Y, Yamamoto Y, Tabuchi H, Fukushima A. Retinal pigment epithelium folds as a diagnostic finding of Vogt-Koyanagi-Harada disease. Japanese journal of ophthalmology. 2013;57(1):90–4. 10.1007/s10384-012-0212-x . 23149670

[6] Nakayama M, Keino H, Okada AA, Watanabe T, Taki W, Inoue M, et al Enhanced depth imaging optical coherence tomography of the choroid in Vogt-Koyanagi-Harada disease. Retina. 2012;32(10):2061–9. 10.1097/IAE.0b013e318256205a . 23095726

[7] Ikewaki J, Kimoto K, Choshi T, Nagata M, Motomura Y, Tamura K, et al Optical coherence tomographic assessment of dynamic macular changes in patients with Vogt-Koyanagi-Harada disease. International ophthalmology. 2011;31(1):9–13. Epub 2011/01/05. 10.1007/s10792-010-9412-x . 21194005

[8] Yamamoto M, Nishijima K, Nakamura M, Yoshimura N. Inner retinal changes in acute-phase Vogt-Koyanagi-Harada disease measured by enhanced spectral domain optical coherence tomography. Japanese journal of ophthalmology. 2011;55(1):1–6. Epub 2011/02/19. 10.1007/s10384-010-0900-3 . 21331684

[9] Lin D, Chen W, Zhang G, Huang H, Zhou Z, Cen L, et al Comparison of the optical coherence tomographic characters between acute Vogt-Koyanagi-Harada disease and acute central serous chorioretinopathy. BMC ophthalmology. 2014;14(1):87 10.1186/1471-2415-14-87 24974016 PMC4099160

[10] Tsujikawa A, Yamashiro K, Yamamoto K, Nonaka A, Fujihara M, Kurimoto Y. Retinal cystoid spaces in acute Vogt-Koyanagi-Harada syndrome. American journal of ophthalmology. 2005;139(4):670–7. Epub 2005/04/06. 10.1016/j.ajo.2004.11.053 . 15808163

[11] Parc C, Guenoun JM, Dhote R, Brezin A. Optical coherence tomography in the acute and chronic phases of Vogt-Koyanagi-Harada disease. Ocular immunology and inflammation. 2005;13(2–3):225–7. 10.1080/09273940490912416 . 16019683

[12] Ishihara K, Hangai M, Kita M, Yoshimura N. Acute Vogt-Koyanagi-Harada disease in enhanced spectral-domain optical coherence tomography. Ophthalmology. 2009;116(9):1799–807. Epub 2009/08/01. 10.1016/j.ophtha.2009.04.002 . 19643489

[13] Lee JE, Park SW, Lee JK, Choi HY, Oum BS, Kim HW. Edema of the photoreceptor layer in Vogt-Koyanagi-Harada disease observed using high-resolution optical coherence tomography. Korean journal of ophthalmology: KJO. 2009;23(2):74–9. Epub 2009/07/02. 10.3341/kjo.2009.23.2.74 19568354 PMC2694296

[14] de Smet MD, Rao NA. Retinal cystoid spaces in acute Vogt-Koyanagi-Harada syndrome. American journal of ophthalmology. 2005;140(5):962; author reply -3. Epub 2005/11/29. 10.1016/j.ajo.2005.06.051 . 16310504

[15] Keane PA, Rao NA, Sadda SR. OCT interpretations. Ophthalmology. 2010;117(3):642, e1; author reply 3, 3 e1. 10.1016/j.ophtha.2009.09.051 . 20189040

[16] Yamaguchi Y, Otani T, Kishi S. Tomographic features of serous retinal detachment with multilobular dye pooling in acute Vogt-Koyanagi-Harada disease. American journal of ophthalmology. 2007;144(2):260–5. Epub 2007/05/30. 10.1016/j.ajo.2007.04.007 . 17533104

[17] Mutlak JA, Dutton GN. Fluorescein angiographic features of acute central serous retinopathy. A retrospective study. Acta Ophthalmol (Copenh). 1989;67(4):467–9. Epub 1989/08/01. . 2801052 10.1111/j.1755-3768.1989.tb01634.x

[18] Ahlers C, Golbaz I, Einwallner E, Dunavolgyi R, Malamos P, Stock G, et al Identification of optical density ratios in subretinal fluid as a clinically relevant biomarker in exudative macular disease. Investigative ophthalmology & visual science. 2009;50(7):3417–24. Epub 2009/01/27. 10.1167/iovs.08-2759 . 19168899

[19] Neudorfer M, Weinberg A, Loewenstein A, Barak A. Differential optical density of subretinal spaces. Investigative ophthalmology & visual science. 2012;53(6):3104–10. Epub 2012/04/14. 10.1167/iovs.11-8700 . 22499985

[20] Chen X, Hou P, Jin C, Zhu W, Luo X, Shi F, et al Quantitative analysis of retinal layer optical intensities on three-dimensional optical coherence tomography. Investigative ophthalmology & visual science. 2013;54(10):6846–51. 10.1167/iovs.13-12062 . 24045992 PMC5963175

[21] Barthelmes D, Sutter FK, Gillies MC. Differential optical densities of intraretinal spaces. Investigative ophthalmology & visual science. 2008;49(8):3529–34. 10.1167/iovs.07-1320 . 18441298

[22] Alasil T, Keane PA, Updike JF, Dustin L, Ouyang Y, Walsh AC, et al Relationship between optical coherence tomography retinal parameters and visual acuity in diabetic macular edema. Ophthalmology. 2010;117(12):2379–86. 10.1016/j.ophtha.2010.03.051 . 20561684 PMC6581779

[23] Horii T, Murakami T, Nishijima K, Akagi T, Uji A, Arakawa N, et al Relationship between fluorescein pooling and optical coherence tomographic reflectivity of cystoid spaces in diabetic macular edema. Ophthalmology. 2012;119(5):1047–55. Epub 2012/02/15. 10.1016/j.ophtha.2011.10.030 . 22330965

[24] Sonoda S, Sakamoto T, Shirasawa M, Yamashita T, Otsuka H, Terasaki H. Correlation between reflectivity of subretinal fluid in OCT images and concentration of intravitreal VEGF in eyes with diabetic macular edema. Investigative ophthalmology & visual science. 2013;54(8):5367–74. 10.1167/iovs.13-12382 . 23860753

